# Objective assessment of cesarean section suturing techniques using a uterine simulator

**DOI:** 10.1038/s41598-026-37041-9

**Published:** 2026-02-05

**Authors:** Hikari Nakato, Jota Maki, Chiaki Kuriyama, Shujiro Sakata, Keiichi Oishi, Ayano Suemori, Hikaru Ooba, Tomohiro Mitoma, Masakazu Kato, Sakurako Mishima, Akiko Ohira, Satoe Kirino, Eriko Eto, Hisashi Masuyama

**Affiliations:** 1 Department of Obstetrics and Gynecology, Okayama University Graduate School of Medicine, Dentistry, and Pharmaceutical Sciences, 2-5-1 Shikata-cho, Kita-ku, Okayama, 700-8558 Japan; 2 Center for Innovative Clinical Medicine, Okayama University Hospital, 2-5-1, Shikata-cho, Kita-ku, Okayama, 700-8558 Japan; 3 Fujihara Ladies Clinic, 5-2-1 Gion Asaminami-ku, Hiroshima, 731-0138 Japan; 4 Division of Epidemiology, Tohoku University Graduate School of Medicine, 2-1 Seiryo-cho, Aoba-ku, Sendai, 980-8575 Japan; 5 Department of Obstetrics and Gynecology, Chugoku Central Hospital, 148-13 Kami-Iwanari, Miyuki-cho, Fukuyama, Hiroshima 720-0001 Japan

**Keywords:** Cesarean section, Simulation, Cesarean scar defects, Barbed suture, Translational research, Therapeutics

## Abstract

Cesarean wound healing is influenced by surgeon experience, suture type, and technique. This study utilized a simulation model to quantify these effects. Obstetricians–gynecologists and junior residents performed two-layer continuous suturing on uterine models, forming eight groups based on experience level (expert, novice), suture type (conventional, barbed), and technique (Albert–Lembert, layer-to-layer). The ideal wound condition was defined as that achieved by an expert using barbed sutures and the layer-to-layer technique. Wound characteristics were quantified and compared to this ideal. Experts using barbed sutures in Albert–Lembert suturing showed higher wound density but greater deformation and larger endometrial openings (both P < 0.01). Novices using barbed sutures in Albert–Lembert suturing showed similar wound density but significantly greater deformation and opening (both P < 0.01). Novices using conventional sutures in layer-to-layer suturing showed the lowest wound density and longest suturing time (both P < 0.01). Notably, novices using barbed sutures achieved wound characteristics comparable to experts using conventional sutures in Albert–Lembert suturing and results closer to the ideal in layer-to-layer suturing. These findings establish a quantifiable standard for cesarean suturing and suggest that optimizing suture types and techniques may help compensate for differences in surgical expertise.

**Supplementary Information:**

The online version contains supplementary material available at 10.1038/s41598-026-37041-9.

## Introduction

Cesarean section rates have increased globally, exceeding 25% of all newborn deliveries in Japan and reaching 50% in some countries^1,2^. This increase has prompted growing concern over cesarean scar defects, which can cause infertility, high-risk pregnancies, and postpartum complications, thereby affecting women’s quality of life^3^. Risk factors for cesarean scar defects include inexperienced surgeons, advanced cervical dilation at the time of cesarean section, and a retroverted uterus^4^.

Previous studies have indicated that suturing techniques, including continuous versus interrupted sutures^5^, single- versus double-layer closure^6^, decidual layer inclusion^7^, and monofilament versus multifilament sutures^8^, may influence the development of cesarean scar defects. Among double-layer uterine closure methods, two different approaches have been described: (1) a technique in which the first layer partially sutures the myometrium followed by approximation of the remaining tissue in the second layer^9,10^, and (2) a technique in which the first layer incorporates the full thickness of the myometrium and the second layer reinforces the closure by approximating the surrounding tissue^10,11^. However, differences in their effects on uterine wound healing remain unclear.　Nevertheless, suturing methods vary widely across regions and institutions. Recent evidence revealed that barbed sutures, compared with conventional sutures may significantly reduce the incidence of cesarean scar defects^9,12^. However, the mechanism by which suture type impacts cesarean scar defects is unclear.

To date, no study has objectively quantified the impact of surgeon skill and suture type on cesarean wound characteristics using in vivo models. Although in vivo studies are limited by practical constraints, simulators offer advantages such as repeated practice, measurement of wound pressure and suture counts, and observation of the wound’s internal surface^13^. Previous studies have successfully quantified both novice and expert suturing techniques using simulators for laparoscopy^14^; however, this approach has not yet been applied to uterine closure during cesarean section.

Therefore, in this study, we aimed to objectively evaluate cesarean wound closure using a uterine model simulator by examining different combinations of surgeon experience, suture type, and suturing technique.

## Results

A total of 40 novices and 30 experts (mean experience 4.1 and 16.8 years; range: 1–8 and 7–35 years, respectively) consented to participate in the study. A table summarizing each surgeon’s years of experience, board certification status in obstetrics and gynecology, presence of subspecialty certification and its details, hospital affiliation, and clinical practice details is provided (Supplemental 1).

Ideal value.

The following measurements were obtained as the ideal values (Table 1, in bold): suture time, 450 (interquartile range: 367–549) s; first layer stitches, 14 (12–15) stitches; second layer stitches, 13 (12–15) stitches; highest pressure, 10.1 (9.3–10.9) hPa; decompression time, 26.2 (7.7–50.2) s; width, 170 (167–171) mm; length, 132 (131–134) mm; endometrial opening area, 79 (47–143) mm^2^. There were eight groups formed from the combinations of two surgeon experience levels, two suture types, and two suturing techniques. Model 1 represented the ideal situation and was compared with other combinations of surgeon experience, suture type, and suturing technique. The remaining seven groups were designated as Models 2 to 8.

**Table 1:** Comparison of ideal values with different combinations of Surgeons, suture Types, and Techniques.

	①Model 1 Ideal value Expert Barbed LL Median (IQR)	②Model 2 Expert Barbed AL	①vs② Padj / *r*	③Model 3 Expert Conventional LL	①vs③ Padj / *r*	④Model 4 Expert Conventional AL	①vs④ Padj / *r*	⑤Model 5 Novice Barbed LL	①vs⑤ Padj / *r*	⑥Model 6 Novice Barbed AL	①vs⑥ Padj / *r*	⑦Model 7 Novice Conventional LL	①vs⑦ Padj / *r*	⑧Model 8 Novice Conventional AL	①vs⑧ Padj / *r*
Suture time (s)	**450** **(367–549)**	464 (379–554)	1/−0.078	601 (505–666)	< 0.001/−0.57	613 (534–756)	< 0.001/−0.62	568 (388–688)	1/−0.18	608 (489–851)	0.13/−0.44	747 (552–994)	< 0.001/−0.62	868 (651–1163)	< 0.001/−0.79
Stitches first layer(stitches)	**14** **(12–15)**	13 (11–14)	0.063/+0.18	15 (13–17)	0.019/−0.27	14 (12–15)	1/−0.013	13 (12–15)	1/+0.16	13 (12–15)	1/+0.13	15 (13–17)	1/−0.22	15 (14–16)	1/−0.28
Stitches second layer(stitches)	**13** **(12–15)**	13 (12–16)	1/−0.027	15 (12–16)	0.24/−0.21	14 (12–16)	1/−0.16	13 (12–15)	1/+0.05	13 (12–15)	1/+0.14	14 (13–16)	1/−0.10	14 (13–16)	1/−0.05
Highest pressure(hPa)	**10.1** **(9.3–10.9)**	12.3 (10.3–13.3)	0.069/−0.42	8.4 (7.0− 9.5)	< 0.001/+0.52	10.2 (9.2–12.5)	1/−0.066	9.7 (8.4–10.5)	1/+0.27	11.1 (9.6–12.3)	1/−0.06	7.9 (7.0−9.3)	0.002/+0.63	10.6 (9.1–12.4)	1/+0.01
Decompression time(s)	**26.2** **(7.7–50.2)**	58.7 (9.4–80.9)	0.011/−0.31	9.2 (3.9–14.7)	< 0.001/+0.46	20.8 (8.8–42.8)	1/+0.068	8.4 (5.4–36.8)	1/+0.46	13.2 (7.7–62.6)	1/+0.21	5.4 (3.3–10.3)	< 0.001/+0.7	11.3 (6.3–30.2)	1/+0.40
Width(mm)	**170** **(167–171)**	174 (170–175)	0.007/−0.55	169 (168–171)	1/+0.007	174 (170–177)	0.006/−0.59	170 (169–173)	1/−0.11	175 (172–178)	0.36/−0.49	170 (169–173)	1/−0.22	175 (173–176)	< 0.001/−0.78
Length(mm)	**132** **(131–134)**	130 (125–131)	< 0.001/+0.61	134 (132–135)	1/−0.23	128 (124–131)	< 0.001/+0.70	133 (132–135)	1/−0.16	129 (125–131)	0.010/+0.58	133 (132–134)	1/−0.21	128 (123–130)	< 0.001/+0.75
Endometrial opening area(mm^2^)	**79** **(47–143)**	560 (238–751)	< 0.001/−0.62	75 (30–156)	1/+0.06	591 (323–858)	< 0.001/−0.83	44 (36–88)	1/+0.26	409 (283–602)	0.002/−0.73	39.4 (12.5–98.6)	1/+0.30	466 (362–633)	< 0.001/−0.86

This table compares ideal values (in bold) with combinations of surgeon experience and suture type and suturing technique. The “ideal value” refers to the wound condition achieved by experts using barbed sutures with the LL technique.

Expert: expert surgeons; Novice: novice surgeons; Barbed: barbed suture; Conventional: conventional suture; LL: layer-to-layer suturing technique; AL: Albert–Lembert suturing technique; Padj: adjusted P-value (Bonferroni correction); r: effect size (rank biserial correlation); IQR: interquartile range.

Comparison of the ideal value (Model 1) with models sutured by experts (Model 2, 3, and 4) revealed significant differences (Table 1). Suture time was significantly shorter in Model 1 than in Models 3 (Padj < 0.001 / *r* = −0.57) and 4 (Padj < 0.001 / *r* = −0.62).

Model 1 required significantly fewer first-layer stitches than Model 3 (Padj < 0.001 / *r* = −0.27). The highest measured pressure was significantly higher in Model 1 than in Model 3 (Padj < 0.001 / r = +0.52). Decompression time was significantly longer in Model 1 than in Models 2 (Padj = 0.011 / r = +0.31) and 3 (Padj < 0.001 / r = +0.46).

The endometrial opening area was significantly smaller in Model 1 than in Models 2 (Padj < 0.001 / *r* = −0.78) and 4 (Padj < 0.001 / *r* = −0.83).

A comparison of the ideal model (Model 1) with novice-sutured models (Models 5–8) is shown in Table 1. Suture time was significantly longer in Models 7 (Padj = 0.016 / r = +0.62) and 8 (Padj < 0.001 / r = +0.79) than in Model 1. There were no significant differences in first-layer stitch counts after adjustment. The highest measured pressure was significantly higher in Model 1 than in Model 7 (Padj < 0.001 / *r* = −0.63) and Model 8 (Padj = 0.003 / *r* = −0.28). Decompression time was significantly shorter in Model 7 than in Model 1 (Padj < 0.001 / *r* = −0.70). The endometrial opening area was significantly larger in Models 6 (Padj = 0.002 / *r* = −0.73) and 8 (Padj < 0.001 / *r* = −0.86) than in Model 1.

The group of experts who used conventional sutures (Models 3 and 4) was compared with the novice group who used barbed sutures (Models 5 and 6; Table 2). When comparing the Albert–Lembert suturing technique (Model 4 vs. Model 6), no statistically significant differences were found after adjustment for multiple testing, indicating comparable surgical performance between the two groups. In contrast, during the layer-to-layer suturing technique (Model 3 vs. Model 5), the novice barbed-suture group required significantly fewer first-layer stitches (Padj = 0.025). Additionally, the highest pressure tended to be higher in this group. Moreover, when comparing the ideal value with Models 3 and Model 5 demonstrated values that were closer to the ideal in terms of suture time, second-layer stitches, highest pressure, width, and length than Model 3.

**Table 2:** Comparison between experts using conventional sutures and novices using barbed sutures.

	④Model 4 Expert Conventional AL Median (IQR)	⑥Model 6 Novice Barbed AL Median (IQR)	Padj / *r*	③Model 3 Expert Conventional LL Median (IQR)	⑤Model 5 Novice Barbed LL Median (IQR)	Padj / *r*
Suture time (s)	613 (534–756)	608 (489–851)	1/+0.15	601 (505–666)	568 (388–688)	1/+0.15
Stitches first layer (stitches)	14 (12–15)	13 (12–15)	1/−0.09	15 (13–17)	13 (12–15)	0.025/+0.35
Stitches second layer (stitches)	14 (12–16)	13 (12–15)	1/−0.19	15 (12–16)	13 (12–15)	1/+0.18
Highest pressure (hPa)	10.2 (9.2–12.5)	11.1 (9.6–12.3)	1/−0.08	8.4 (7.0–9.5)	9.7 (8.4–10.5)	0.08/−0.31
Decompression time (s)	20.8 (8.8–42.8)	13.2 (7.7–62.6)	1/+0.27	9.2 (3.9–14.7)	8.4 (5.4–36.8)	1/−0.17
Width (mm)	174 (170–177)	175 (172–178)	1/−0.13	169 (168–171)	170 (169–173)	1/+0.03
Length (mm)	128 (124–131)	129 (125–131)	1/+0.15	134 (132–135)	133 (132–135)	1/+0.12
Endometrial opening area (mm^2^)	591 (323–858)	409 (283–602)	1/−0.18	75 (30–156)	44 (36–88)	1/+0.18

Expert: expert surgeons; Novice: novice surgeons; Barbed: barbed sutures; Conventional: conventional sutures;

LL: layer-to-layer suturing technique; AL: Albert–Lembert suturing technique;

Padj: adjusted P-value (Bonferroni correction); r: effect size (rank biserial correlation); IQR: interquartile range.

## Discussion

Using a uterine simulator, significant differences in cesarean section wound conditions were observed based on surgeon experience, suture type, and technique. In Albert–Lembert suturing, novices using barbed sutures achieved results comparable to experts using conventional sutures, In contrast, in layer-to-layer suturing, novices using barbed sutures showed a significantly lower number of first-layer stitches and a tendency toward higher highest pressure compared with experts using conventional sutures. Furthermore, they also demonstrated results closer to the ideal value in terms of suturing time, number of second-layer stitches, width and length.

This study revealed that surgeon experience, suture type, and suturing technique combination significantly affect wound condition; however, the effect on wound healing remains unclear. Appropriate suture tension, spacing, and wound-edge alignment are essential for wound healing, and improper application can lead to impaired blood flow, chronic inflammation, and scarring^15–17^. Histopathological evaluation of cesarean section wounds has shown that early blood flow impairment and necrosis can contribute to scar formation^15,18^.

Although insights into suture spacing and tension exist for skin and intestinal sutures^19–22^, their effects on the postpartum contracting uterus remain unknown. A study using a simulator with contracting materials for two-layer continuous suturing reported higher wound dehiscence with conventional sutures compared with barbed sutures^23^, emphasizing the importance of appropriate suture materials and techniques to prevent uterine scarring.

The relative effectiveness of Albert–Lembert versus layer-to-layer techniques in preventing cesarean scar defects remains uncertain in clinical settings. In the present simulator-based evaluation, Models 2, 4, 6, and 8 showed that the Albert–Lembert technique significantly increased the area of endometrial opening and deformation, as well as wound density (highest pressure and decompression time), which are surrogate mechanical parameters and do not have established clinical correlation. These findings may indicate that excessive suture density could increase mechanical stress on the tissue. Barbed sutures reduce operator traction, closely approximate the myometrium, and eliminate knot-tying, which may theoretically lessen mechanical trauma^24,25^. However, the combination of barbed sutures and the Albert–Lembert technique increased wound density compared with conventional sutures in Models 2 and 6. Taken together, the layer-to-layer technique combined with barbed sutures may theoretically contribute to more favorable immediate wound characteristics in a simulated setting; however, this has not been demonstrated in vivo, and clinical implications should be interpreted cautiously.

Novice-performed Albert–Lembert suturing resulted in lower wound density than expert-performed suturing using the same technique, regardless of suture type, with comparable areas of endometrial opening to Models 2 and 4. This may be due to insufficient myometrial adhesion by novices, as observed in Models 6 and 8. Oversized bites in the second layer create inward and outward vectors on the wound, increasing endometrial opening, while excessively small bites risk myometrial rupture, highlighting the need for precise bite size and advanced techniques.

Skill acquisition requires extensive training, and long-term practice is essential for expertise. Advances in surgical techniques and reduced training opportunities highlight the importance of simulation-based education^26^. Abdullatif et al.^27^ conducted a 10-country randomized clinical trial on simulator-based training in urological surgeries, and demonstrated faster proficiency and fewer complications in simulator-trained groups. High-fidelity simulators, like those developed by Uemura et al.^14^, quantify suturing quality and support rapid skill improvement.

Approximately 10 to 40 supervised cesarean section procedures are recommended before independent practice^27^. The uterine model simulator in this study enabled repeated practice without burdening patients, promoting skill acquisition and early independence through quantifiable metrics. Novices using barbed sutures could achieve or surpass outcomes of experts using conventional sutures with either technique, suggesting that barbed sutures are suitable for novices and help experts achieve optimal wound conditions.

This study quantified the immediate condition of cesarean wounds using a uterine model simulator; because the simulator does not reproduce uterine contractions or vascular perfusion, the results may not fully represent the biomechanical conditions or postoperative healing processes in actual cesarean wounds. Future research using biological models is needed to evaluate post-cesarean wound conditions in real uteri and the potential effects of uterine contractions. Surgeon performance may have been influenced by familiarity with specific suture materials and techniques, as well as by skill improvement through repeated practice, since each surgeon consecutively sutured four uterine models. Because the suturing order was not randomized, potential learning effects could not be fully excluded. The combination defined as ideal in this study is based on previously reported surgical methods; however, further investigation is required. Additionally, measurements were taken in bulk after accumulating approximately 20 sutured sheets, which may have allowed suture hydrolysis to affect the results. Although each measurement item was evaluated by a single operator to maintain consistency, intra- and inter-observer variability were not assessed, which may affect the reliability of the measurements and represents a limitation of this study. Furthermore, because multiple comparisons were conducted across several outcomes and groups, the risk of type I error may not be fully eliminated, even though Bonferroni correction and effect sizes were applied to reduce this risk. The current study did not evaluate whether training with the uterine model simulator improves performance in actual cesarean sections or affects the incidence of complications. Although a priori power analysis was not conducted, the sample size was determined based on a previous simulator study, and we included more participants to maintain adequate group sizes. This remains a limitation of the study.

We successfully quantified ideal suturing conditions in the uterine model, enabling the creation of a standard suturing level. Furthermore, the study indicated that adjustments in sutures and suturing techniques could potentially compensate for differences in expertise.

## Materials and methods

### Study design and participants

This experimental study was approved by the Research Ethics Committee of Okayama University School of Medicine and was conducted between September 2023 and June 2024. All methods were carried out in accordance with relevant guidelines and regulations, including the Declaration of Helsinki. Written informed consent was obtained from all subjects involved in the study. Participants included residents and obstetricians from the university hospital and seven affiliated institutions. Surgeons were classified as “experts” if they had ≥ 6 years of experience and were board-certified obstetricians, while others were considered “novices.” Notably, certification as a board-certified obstetrician and gynecologist in the country of study requires performing at least 30 cesarean sections as the primary surgeon and participating in at least 100 vaginal deliveries. To determine the sample size for this simulation-based study, we referred to a previous investigation that objectively evaluated suturing skills using a laparoscopic intestinal anastomosis simulator^14^. In that study, 36 novices and 17 experts were enrolled. Considering that laparoscopic bowel suturing is technically more demanding than open suturing in cesarean delivery and therefore may produce greater performance differences, we increased the number of participants to ensure adequate group sizes in the current study, including 40 novices and 30 experts. Although a priori power analysis was not conducted, this point has been acknowledged as a limitation in the Discussion.

### Uterine model and suture

The uterine model consisted of a uniform-density elastic resin pad measuring 162 × 151 mm and 160 × 150 mm, respectively, when laid flat and when fixed at four points, with a middle 100 mm incision (Supplemental 2). The incision size was comparable to that of typical cesarean incisions^9^. Two types of sutures were used including, conventional suture (0-Vicryl [polyglactin 910; JMDN Code:17471000; 15700BZY01341000; Ethicon, Johnson & Johnson])^28^ and barbed suture (0-STRATAFIX Spiral PDS Plus [polydioxanone; JMDN Code: 16584000; 22900BZX00123000; Ethicon, Johnson & Johnson])^29^.

### Assessment task and objective data collection

The uterine model was mounted on a platform tilted at 20° to simulate a late gravid uterus. The uterine model was fixed at two upper and three lower points. The model was oriented with the upper side toward the patient’s head and the lower side toward the tail end (Supplemental 3).

**Fig. 1:**
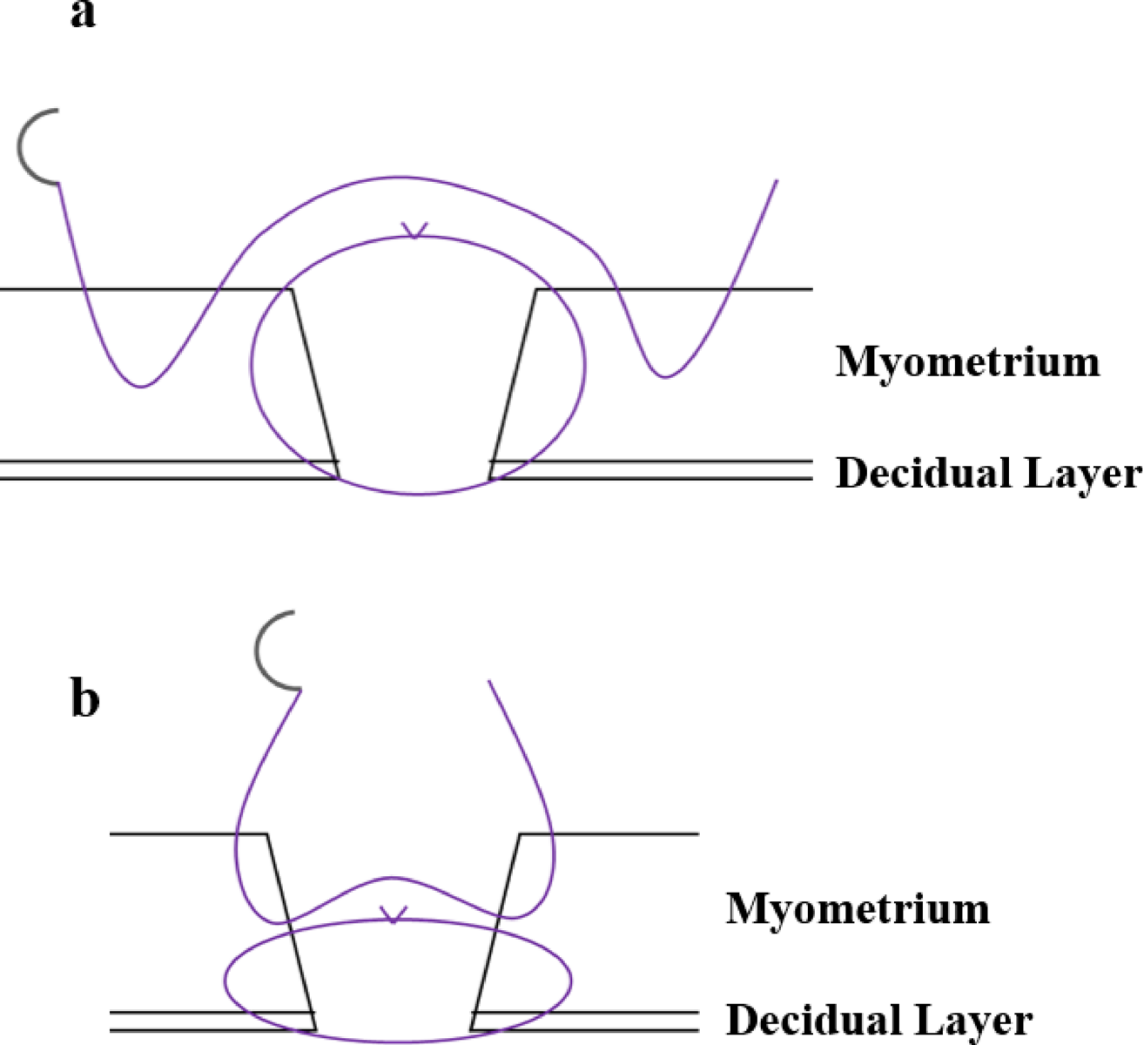
Suture technique. Albert–Lembert suture technique: The first layer closes the full-thickness uterine incision, whereas the second layer approximates the myometrium to reduce tension. Layer to Layer suture technique: Approximately one-fourth of the muscle layer on the peritoneal side remains untouched, with the second layer covering the sutures of the first layer.

The surgeons performed a two-layer continuous closure using the two different sutures and two suturing techniques– the Albert–Lembert and layer-to-layer methods (Fig. 1). In the Albert–Lembert technique, the first layer closes the full thickness of the uterine incision, whereas the second layer approximates the myometrium to reduce tension^10,11^. In the layer-to-layer technique, approximately one-fourth of the muscle layer on the peritoneal side is untouched, with the second layer covering the first layer’s sutures^9,10^. Conventional sutures require knot tying at the beginning and end of suturing. However, for the barbed sutures, a single reverse stitch was performed at the start and two reverse stitches at the end, opposite the direction of the needle passage to secure the suture. We instructed all surgeons on the suturing method before the procedure. The decidua was included in the closure in both suturing techniques. We addressed the potential for practice-related skill improvement bias, considering that each participant performed four suturing procedures, by ensuring that participants initially used the suture materials and techniques they were accustomed to using in their routine practice. All suturing procedures were video-recorded for subsequent analysis. Each measurement item was evaluated consistently by a single trained operator to minimize variability.

### Five suturing skills assessment (Fig. 2)

**Fig. 2:**
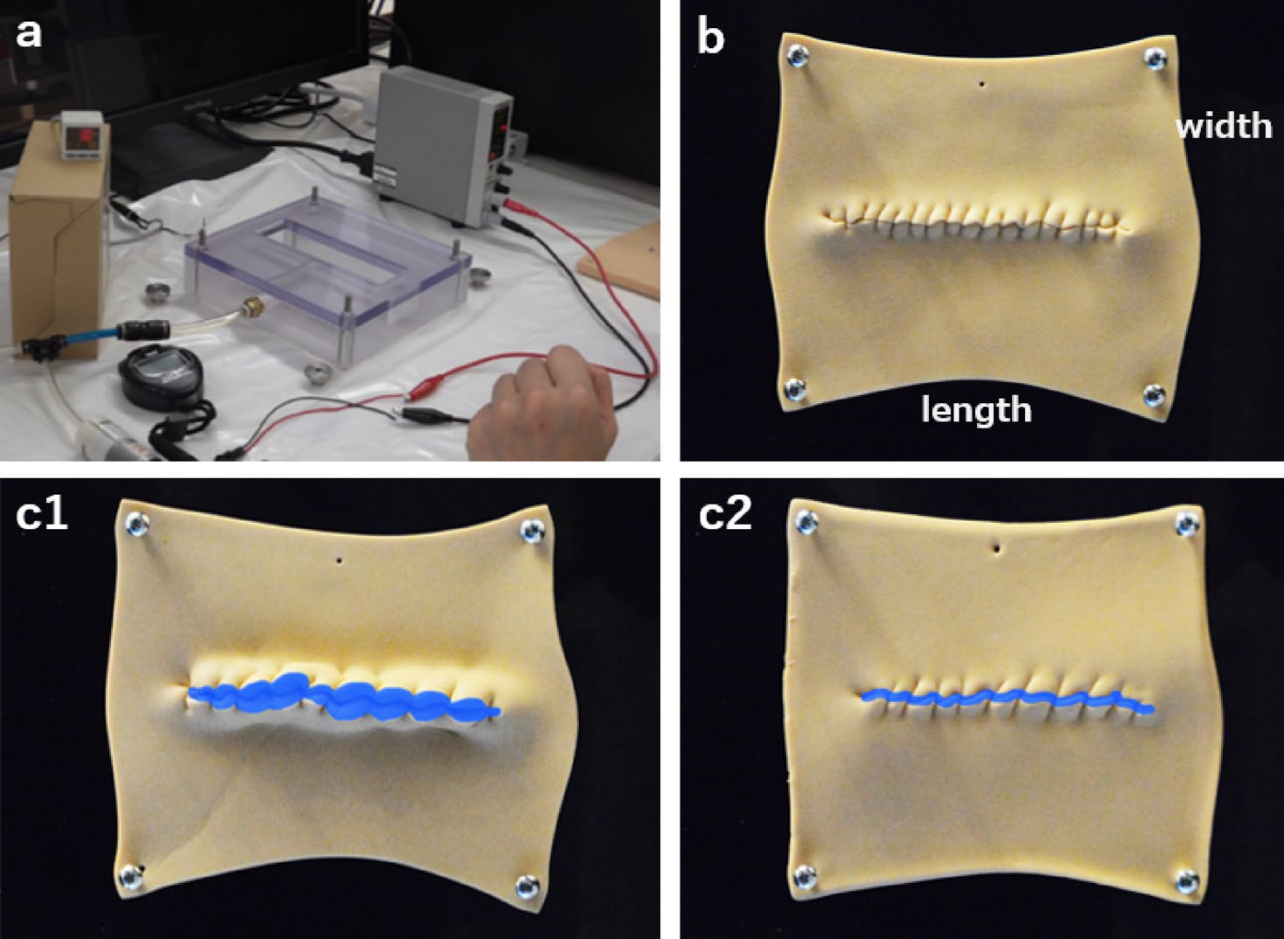
Measurement method. (**a**) Device for measuring wound density (highest pressure [hPa] and decompression time [s]). The measurement method is shown in Supplementary Video 1. (**b**) Method for measuring model deformation (length and width [mm]). (**c**) Endometrial opening area (mm^2^) measured from the posterior side. c1 represents the Albert–Lembert suture technique, and c2 represents the layer-to-layer suture technique.

- Suture time (s)
  The time from the start to the completion of suturing was measured.
- Number of stitches (first and second layers).
  Conventional sutures were counted in full, whereas reverse stitches were excluded in the count for barbed suture use.
- Wound density (highest pressure [hPa] and decompression time [s])
  The wound density was measured using a pressure device similar to that described by Uemura et al.^14^ Air was introduced with the sutured uterine model secured at four points on a pressure device and stabilized by an acrylic plate. Subsequently, the maximum air pressure was recorded as the highest pressure (hPa) after initiating the air supply. The time taken for the wound pressure to decrease to 3 hPa was measured as the decompression time (s) after halting the air supply. The highest pressure (hPa) and decompression time (s) were recorded, with the measurements repeated an average of five times. This process is illustrated in Supplemental 4.
- Model deformation (length and width [mm])
  The sutured uterine model was fixed at four points, and its length and width were measured.
- Endometrial opening area (mm^2^).
  The endometrial opening area, as observed from the reverse side, was stained with blue ink and subsequently analyzed using Adobe Photoshop (version 25.11; Adobe Inc., San Jose, CA, USA).
  Each measurement item was evaluated consistently by a single trained operator to minimize variability.

### Ideal value definition

**Fig. 3:**
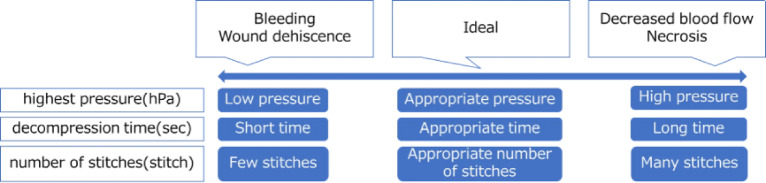
Ideal wound state. Indicate the ideal wound state in the figure.

Impaired blood flow or tissue weakening during the wound healing process can lead to chronic inflammation and scar formation^19^, and excessive suture tension or improper spacing may further compromise blood flow^19,20^. Therefore, adhesive-only approximation of the wound edges (Fig. 3), which does not impose suture-related tension or tissue trauma, would theoretically provide the most ideal wound condition. However, such closure is not clinically feasible.

Accordingly, based on previous literature^4,8,9,12,23^, we defined the wound condition achieved by expert surgeons using barbed sutures with the layer-to-layer technique as the “Ideal Value”, representing the best clinically achievable condition in this study.

### Statistical analysis

Statistical analyses were conducted using Stata/SE (version 18.0; StataCorp, LLC). The Wilcoxon rank-sum test was used for all comparisons. To adjust for multiple testing across groups and outcome measures, Bonferroni correction was applied, and both unadjusted and adjusted P-values (Padj) were reported. Effect sizes (r) were calculated to evaluate the magnitude of differences. Statistical significance was set at P < 0.05. Values were presented as medians and interquartile ranges (IQR) unless otherwise specified.

## Supplementary Information

Below is the link to the electronic supplementary material.

Supplementary Material

Supplementary Material 2

## Data Availability

De-identified data will be shared for research purposes upon reasonable request.The datasets are available from the corresponding author upon request.

## References

[1] Ministry of Health, Labour and Welfare. Health statistics of Japan (business and processing statistics) 2, trends in medical facilities 2016. *Ministry of Health, Labour and Welfare*. https://www.mhlw.go.jp/toukei/list/dl/130-28_2.pdf (Accessed 28 Nov 2024).

[2] Yu, S. M. Healthy people 2010. *Matern Child. Health J.* **2**, 63–66. 10.1023/a:1021801927353 (1998). 10728261 10.1023/a:1021801927353

[3] Tulandi, T. & Cohen, A. Emerging manifestations of cesarean scar defect in reproductive-aged women. *J. Minim. Invasive Gynecol.* **23**, 893–902. 10.1016/j.jmig.2016.06.020 (2016). 27393285 10.1016/j.jmig.2016.06.020

[4] Bij de Vaate, A. J. M. et al. Prevalence, potential risk factors for development and symptoms related to the presence of uterine niches following cesarean section: systematic review. *Ultrasound Obstet. Gynecol.* **43**, 372–382. 10.1002/uog.13199 (2014). 23996650 10.1002/uog.13199

[5] Dodd, J. M. et al. Surgical techniques for uterine incision and uterine closure at the time of caesarean section. *Cochrane Database Syst. Rev.* **2014**, CD004732. 10.1002/14651858.CD004732.pub3 (2014). 10.1002/14651858.CD004732.pub3 PMC11182567 25048608

[6] Di Sardo, S. Risk of cesarean scar defect following single- vs double-layer uterine closure: systematic review and meta-analysis of randomized controlled trials. *Ultrasound Obstet. Gynecol.* **50**, 578–583. 10.1002/uog.17401 (2017). 28070914 10.1002/uog.17401

[7] Stegwee, S. I. et al. Uterine caesarean closure techniques affect ultrasound findings and maternal outcomes: a systematic review and meta-analysis. *BJOG* **125**, 1097–1108. 10.1111/1471-0528.15048 (2018). 29215795 10.1111/1471-0528.15048

[8] Başbuğ, A., Doğan, O., Ellibeş Kaya, A., Pulatoğlu, Ç. & Çağlar, M. Does suture material affect uterine scar healing after cesarean section? Results from a randomized controlled trial. *J. Invest. Surg.* **32**, 763–769. 10.1080/08941939.2018.1458926 (2019). 29667541 10.1080/08941939.2018.1458926

[9] Maki, J. et al. Barbed vs conventional sutures for cesarean uterine scar defects: a randomized clinical trial. *Am. J. Obstet. Gynecol. MFM*. **6**, 101431. 10.1016/j.ajogmf.2024.101431 (2024). 39019212 10.1016/j.ajogmf.2024.101431

[10] Takeda, Y. Management of childbirth: cesarean section. *Perinat. Med.* **54**, 232–234 (2024).

[11] Stegwee, S. I. et al. Single- versus double-layer closure of the caesarean scar in the prevention of gynaecological symptoms in relation to niche development: the 2Close study. *BMC Pregnancy Childbirth*. **19**, 85. 10.1186/s12884-019-2221-y (2019). 30832681 10.1186/s12884-019-2221-y PMC6399840

[12] Alessandri, F. et al. Incidence and ultrasonographic characteristics of cesarean scar niches after uterine closure by double-layer barbed suture: a prospective comparative study. *Int. J. Gynaecol. Obstet.* **162**, 895–905. 10.1002/ijgo.14744 (2023). 36825332 10.1002/ijgo.14744

[13] Bjerrum, F., Thomsen, A. S. S., Nayahangan, L. J. & Konge, L. Surgical simulation: current practices and future perspectives for technical skills training. *Med. Teach.* **40**, 668–675. 10.1080/0142159X.2018.1472754 (2018). 29911477 10.1080/0142159X.2018.1472754

[14] Uemura, M. et al. Objective assessment of the suture ligature method for the laparoscopic intestinal anastomosis model using a new computerized system. *Surg. Endosc*. **29**, 444–452. 10.1007/s00464-014-3681-913 (2015). 25005015 10.1007/s00464-014-3681-9

[15] Sorg, H., Tilkorn, D. J., Hager, S., Hauser, J. & Mirastschijski, U. Skin wound healing: an update on the current knowledge and concepts. *Eur. Surg. Res.* **58**, 81–94. 10.1159/000454919 (2017). 27974711 10.1159/000454919

[16] Wang, P. H., Huang, B. S., Horng, H. C., Yeh, C. C. & Chen, Y. J. Wound healing. *J. Chin. Med. Assoc.* **81**, 94–101. 10.1016/j.jcma.2017.11.002 (2018). 29169897 10.1016/j.jcma.2017.11.002

[17] Lin, X. & Lai, Y. Scarring skin: mechanisms and therapies. *Int. J. Mol. Sci.* **25**, 1458. 10.3390/ijms25031458 (2024). 38338767 10.3390/ijms25031458 PMC10855152

[18] Morris, H. Surgical pathology of the lower uterine segment caesarean section scar: is the scar a source of clinical symptoms? *Int. J. Gynecol. Pathol.* **14**, 16–20. 10.1097/00004347-199501000-00004 (1995). 7883420 10.1097/00004347-199501000-00004

[19] Oguma, J. et al. Knot-tying force during suturing and wound healing in the gastrointestinal tract. *J. Surg. Res.* **140**, 129–134. 10.1016/j.jss.2006.12.008 (2007). 17418872 10.1016/j.jss.2006.12.008

[20] Stoecker, A. et al. Effect of simple interrupted suture spacing on aesthetic and functional outcomes of skin closures. *J. Cutan. Med. Surg.* **23**, 580–585. 10.1177/1203475419861077 (2019). 31272216 10.1177/1203475419861077

[21] Sklar, L. R. et al. Comparison of running cutaneous suture spacing during linear wound closures and the effect on wound cosmesis of the face and neck: a randomized clinical trial. *JAMA Dermatol.* **155**, 321–326. 10.1001/jamadermatol.2018.5057 (2019). 30649154 10.1001/jamadermatol.2018.5057 PMC6439934

[22] Eshagh, K. et al. Interrupted subcuticular suture spacing during linear wound closures and the effect on wound cosmesis: a randomized evaluator-blinded split-wound comparative effectiveness trial. *Br. J. Dermatol.* **187**, 318–323. 10.1111/bjd.21625 (2022). 35474448 10.1111/bjd.21625

[23] Maki, J. & Masuyama, T. The role of barbed sutures in reducing uterine scar thinning: cesarean scar reconstruction without knots. *Jpn. J. Perinat. Neonatal Med.* **59**, 479–481 (2024).

[24] Lin, Y., Lai, S., Huang, J. & Du, L. The efficacy and safety of knotless barbed sutures in the surgical field: a systematic review and meta-analysis of randomized controlled trials. *Sci. Rep.* **6**, 23425. 10.1038/srep23425 (2016). 27005688 10.1038/srep23425 PMC4804241

[25] Shiga, T., Okada, H., Isobe, M. & Furui, T. Tissue damage between barbed suture and conventional sutures in animal laboratory model using scanning electron microscopy. *J. Obstet. Gynaecol.* **44**, 2370973. 10.1080/01443615.2024.2370973 (2024). 38934494 10.1080/01443615.2024.2370973

[26] Haiser, A. et al. A systematic review of simulation-based training in vascular surgery. *J. Surg. Res.* **279**, 409–419. 10.1016/j.jss.2022.05.009 (2022). 35839575 10.1016/j.jss.2022.05.009 PMC9483723

[27] Madsen, K. et al. Educational strategies in performing cesarean section. *Acta Obstet. Gynecol. Scand.* **92**, 256–263. 10.1111/aogs.12055 (2013). 23173712 10.1111/aogs.12055

[28] Ethicon Sept. Coated VICRYL™ (polyglactin 910) suture. https://www.jnjmedtech.com/en-US/product/coated-vicryl-polyglactin-910-suture (Accessed 22 Sept 2023).

[29] Ethicon Anatomy of a barbed suture: a comprehensive guide. https://www.jnjmedtech.com/libraries/pdf.js/web/viewer.html?file=/sites/default/files/user_uploaded_assets/pdf_assets/2020-11/STRATAFIX%20Anatomy%20of%20a%20Barbed%20Suture%20Brochure%20081792-171005.pdf#pagemode=none (Accessed 13 June 2023).

