# Supplementary material for: Objective assessment of cesarean section suturing techniques using a uterine simulator
Source: Sci Rep. 2026 Feb 5;16:7456. doi: 10.1038/s41598-026-37041-9 (PMC12929746; doi:10.1038/s41598-026-37041-9)
Supplement: Supplementary file 1 — Supplementary Material [file 41598_2026_37041_MOESM1_ESM.pdf]

Supplemental 1

Supplementary table1: Surgeon details

No.	Years of clinical experience	OBGYN-S (JSOG)	Subspecialty Certifications in OB/GYN in Japan	Affiliated hospital	Clinical Practice Areas
1	6	No	No	University hospital	Gen OB/GYN
2	13	Yes	PMFS (JSPNM)	University hospital	Perinatal Care
3	3	No	No	University hospital	Gen OB/GYN
4	3	No	No	University hospital	Gen OB/GYN
5	3	No	No	University hospital	Gen OB/GYN
6	4	No	No	University hospital	Gen OB/GYN
7	4	No	No	University hospital	Gen OB/GYN
8	4	No	No	University hospital	Gen OB/GYN
9	15	Yes	SRM (JSRM), CLS (JSGOE)	University hospital	REI
10	3	No	No	University hospital	Gen OB/GYN
11	3	No	No	University hospital	Gen OB/GYN
12	3	No	No	University hospital	Gen OB/GYN
13	5	No	No	University hospital	Gen OB/GYN
14	5	No	No	University hospital	Gen OB/GYN
15	5	No	No	University	Gen OB/GYN

				hospital	
16	5	No	No	University hospital	Gen OB/GYN
17	3	No	No	University hospital	Gen OB/GYN
18	6	No	No	University hospital	Gen OB/GYN
19	5	No	No	University hospital	Gen OB/GYN
20	3	No	No	University hospital	Gen OB/GYN
21	15	Yes	PMFS (JSPNM)	University hospital	Perinatal Care
22	12	Yes	PMFS (JSPNM)	University hospital	Perinatal Care
23	7	Yes	No	University hospital	Perinatal Care
24	5	No	No	University hospital	Gen OB/GYN
25	10	Yes	No	University hospital	Perinatal Care
26	8	Yes	No	University hospital	Perinatal Care, Urogynecology
27	13	Yes	PMFS (JSPNM), SWH (JSMWH)	University hospital	Perinatal Care, Urogynecology
28	6	No	No	University hospital	Gen OB/GYN
29	13	Yes	SRM (JSRM)	University hospital	REI
30	5	No	No	University hospital	Gen OB/GYN
31	7	Yes	No	University hospital	Gyn Onc
32	19	Yes	PMFS (JSPNM)	University hospital	Perinatal Care

33	6	No	No	University hospital	Gen OB/GYN
34	6	No	No	University hospital	Gen OB/GYN
35	15	Yes	GOS (JSGO)	University hospital	Gyn Onc
36	7	No	No	University hospital	Gen OB/GYN
37	24	Yes	No	Community hospital	Gen OB/GYN
38	15	Yes	PMFS (JSPNM)	Community hospital	Gen OB/GYN
39	8	Yes	No	Community hospital	Gen OB/GYN
40	7	No	No	University hospital	Gyn Onc
41	7	Yes	No	Community hospital	Perinatal Care
42	32	Yes	GOS (JSGO), CLS (JSGOE)	University hospital	Gyn Onc
43	31	Yes	GOS (JSGO)	University hospital	Gyn Onc
44	2	No	No	University hospital	PGY-2 Resident
45	2	No	No	University hospital	PGY-2 Resident
46	2	No	No	University hospital	PGY-2 Resident
47	3	No	No	University hospital	Gen OB/GYN
48	3	No	No	University hospital	Gen OB/GYN
49	14	Yes	GOS (JSGO)	University hospital	Gyn Onc
50	31	Yes	SRM (JSRM), CLS (JSGOE),	University hospital	REI

			SWH (JSMWH)		
51	27	Yes	No	Community hospital	Gen OB/GYN
52	3	No	No	University hospital	Gen OB/GYN
53	8	No	No	Community hospital	Gen OB/GYN
54	16	Yes	SWH (JSMWH)	Community hospital	Gen OB/GYN
55	33	Yes	No	Community hospital	Gen OB/GYN
56	10	Yes	No	University hospital	Perinatal Care
57	7	No	No	University hospital	Gyn Onc
58	1	No	No	University hospital	PGY-1 Resident
59	2	No	No	University hospital	PGY-2 Resident
60	2	No	No	Community hospital	PGY-2 Resident
61	14	Yes	GOS (JSGO), SRM (JSRM)	University hospital	Gyn Onc
62	35	Yes	SWH (JSMWH)	Community hospital	Gen OB/GYN
63	8	Yes	No	Community hospital	Gen OB/GYN
64	2	No	No	Community hospital	PGY-2 Resident
65	9	Yes	No	University hospital	Gyn Onc
66	2	No	No	Community hospital	PGY-2 Resident
67	2	No	No	Community hospital	PGY-2 Resident

68	16	Yes	PMFS (JSPNM), SWH (JSMWH)	University hospital	REI
69	22	Yes	GOS (JSGO)	University hospital	Gyn Onc
70	12	Yes	PMFS (JSPNM)	Community hospital	Gen OB/GYN

The surgeon's years of experience, possession of an obstetrics and gynecology specialist certification, presence of subspecialty certifications in obstetrics and gynecology (including specific certifications held), hospital affiliation, and clinical practice details were recorded.

OBGYN-S (JSOG): Obstetrics and Gynecology Specialist, Japan Society of Obstetrics and Gynecology

PMFS (JSPNM): Perinatal (Maternal-Fetal) Specialist, Japan Society of Perinatal and Neonatal Medicine

GOS (JSGO): Gynecologic Oncology Specialist, Japan Society of Gynecologic Oncology

SRM (JSRM): Specialist in Reproductive Medicine, Japan Society for Reproductive Medicine

SWH (JSMWH): Specialist in Women's Healthcare, Japan Society for Menopause and Women's Health

CLS (JSGOE): Certified Laparoscopic Surgeon, Japan Society of Gynecologic and Obstetric Endoscopy and Minimally Invasive Therapy

Gen OB/GYN: General Obstetrics and Gynecology

Gyn Onc: Gynecologic Oncology

REI: Reproductive Endocrinology and Infertility

PGY: Postgraduate Year

Supplemental 2

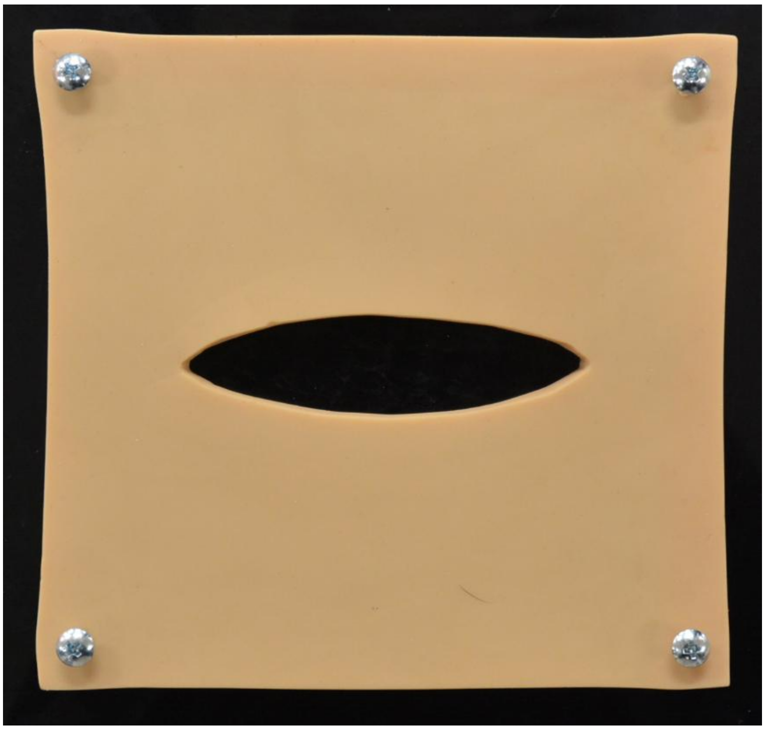

The uterine model measured 162 × 151 and 160 × 150 mm, respectively, when laid flat and when fixed at four points, with a 100-mm incision in the center.

Supplemental 3

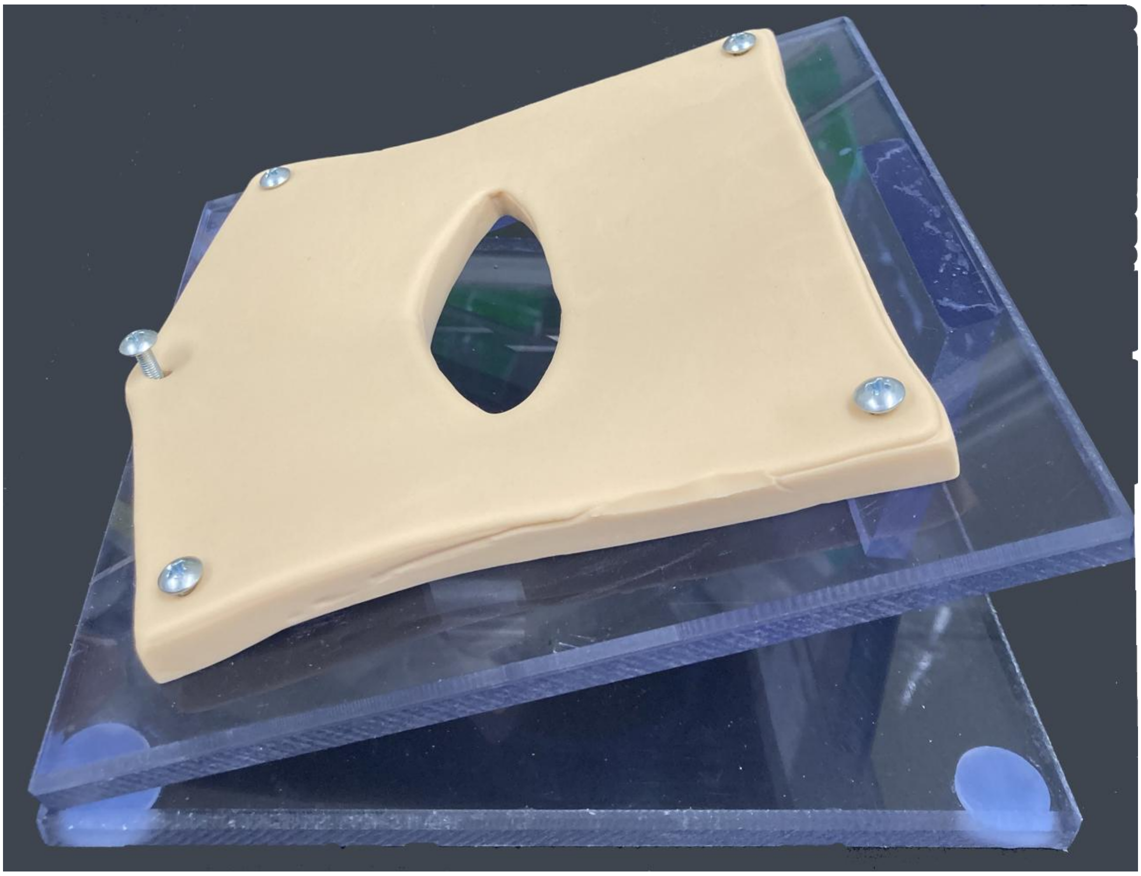

The uterine model was tilted at 20° to mimic a late gravid uterus and fixed at five points, with the upper side oriented toward the patient's head.

Supplemental 4: Video

Measurement of the highest pressure and decompression time: The sutured uterine model was secured at four points on a pressure device and stabilized using an acrylic plate. Air was introduced to record the peak pressure (hPa) and the time for the pressure to drop to 3 hPa after stopping the air supply (s). The measurements were repeated an average five times.

Supplemental 5

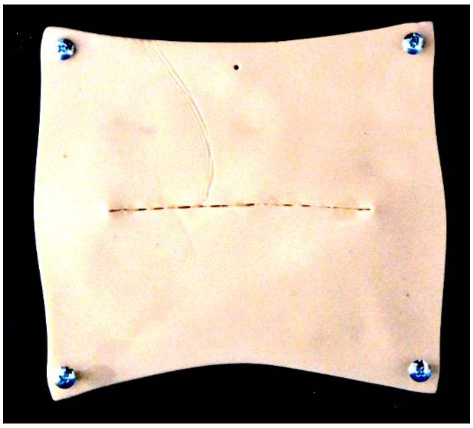

A uterine model with wound edges adhered using glue measuring 168 × 135 cm.
